# Correction: Intravenous thrombolysis for acute central retinal artery occlusion: Protocol for a systematic review and individual participant data meta-analysis of randomized controlled trials

**DOI:** 10.1371/journal.pone.0351934

**Published:** 2026-06-16

**Authors:** Brian Mac Grory, Cécile Preterre, Pierre Lebranchu, Stephen James Ryan, Øystein Kalsnes Jørstad, Morten Carstens Moe, Johannes Tünnerhoff, Martin S. Spitzer, Carsten Grohmann, Oana M. Dumitrascu, Valérie Biousse, Benoit Guillon, Anne Hege Aamodt, Sven Poli, Matthew Schrag, The Central Retinal ArterY OcclusioN (CRAYON) Collaborators

The fifth author’s initials appear incorrectly in the citation. The correct citation is: Mac Grory B, Preterre C, Lebranchu P, Ryan SJ, Kalsnes Jørstad ØK, Moe MC, et al. (2026) Intravenous thrombolysis for acute central retinal artery occlusion: Protocol for a systematic review and individual participant data meta-analysis of randomized controlled trials. PLoS One 21(3): e0342739. https://doi.org/10.1371/journal.pone.0342739.
