# Correction: Correction: Intravenous thrombolysis for acute central retinal artery occlusion: Protocol for a systematic review and individual participant data meta-analysis of randomized controlled trials

**DOI:** 10.1371/journal.pone.0353253

**Published:** 2026-07-06

**Authors:** The PLOS One Staff

The first and fifth author’s initials appear incorrectly in the correction citation posted on June 16, 2026. The correct citation is: Mac Grory B, Preterre C, Lebranchu P, Ryan SJ, Jørstad ØK, Moe MC, et al. (2026) Intravenous thrombolysis for acute central retinal artery occlusion: Protocol for a systematic review and individual participant data meta-analysis of randomized controlled trials. PLoS One 21(3): e0342739. https://doi.org/10.1371/journal.pone.0342739. The publisher apologizes for the error.
